# Efficacy and safety of *Bacteroides fragilis* BF839 for pediatric autism spectrum disorder: a randomized clinical trial

**DOI:** 10.3389/fnut.2024.1447059

**Published:** 2024-09-03

**Authors:** Chu-hui Lin, Ting Zeng, Cui-wei Lu, De-yang Li, Yi-ying Liu, Bing-mei Li, Sheng-qiang Chen, Yu-hong Deng

**Affiliations:** ^1^Department of Clinical Nutrition, The Second Affiliated Hospital of Guangzhou Medical University, Guangzhou, China; ^2^Medical Administration College, Guangzhou Medical University, Guangzhou, China; ^3^Institute of Psychology, Chinese Academy of Sciences, Beijing, China; ^4^Weierkang Specialist Outpatient Department, Guangzhou, China; ^5^Department of Neurology, Institute of Neuroscience, The Second Affiliated Hospital of Guangzhou Medical University, Guangzhou, China; ^6^Key Laboratory of Neurogenetics and Channelopathies of Guangdong Province and the Ministry of Education of China, Guangzhou, China

**Keywords:** autism spectrum disorder in children, probiotics, *Bacteroides fragilis* BF839, abnormal behavior, gastrointestinal symptoms

## Abstract

**Background:**

The clinical utility of *Bacteroides fragilis* in treating autism spectrum disorder (ASD) remains unclear. Therefore, this randomized, double-blind, placebo-controlled study aimed to explore the therapeutic effects and safety of *B. fragilis* BF839 in the treatment of pediatric ASD.

**Methods:**

We examined 60 children aged 2–10 years diagnosed with ASD, and participants received either BF839 powder (10 g/bar with ≥10^6^ CFU/bar of viable bacteria, two bars/day) or placebo for 16 weeks. The primary outcomes was Autism Behavior Checklist (ABC) score. The secondary outcomes were Childhood Autism Rating Scale (CARS), Social Responsiveness Scale (SRS), Normal Development of Social Skills from Infants to Junior High School Children (S-M), Gastrointestinal Symptom Rating Scale (GSRS) scores, and fecal microbiome composition. Assessments were performed on day 0 and at weeks 8 and 16.

**Results:**

Compared with the placebo group, the BF839 group showed significant improvement in the ABC body and object use scores at week 16, which was more pronounced in children with ASD aged <4 years. Among children with a baseline CARS score ≥30, the BF839 group showed significant improvements at week 16 in the ABC total score, ABC body and object use score, CARS score, and GSRS score compared to the placebo group. Only two patients (6.67%) in the BF839 group experienced mild diarrhea. Compared with baseline and placebo group levels, the BF839 group showed a significant post-intervention increase in abundance of *bifidobacteria* and change in the metabolic function of neuroactive compounds encoded by intestinal microorganisms.

**Conclusion:**

BF839 significantly and safely improved abnormal behavior and gastrointestinal symptoms in children with ASD.

## 1 Introduction

Autism spectrum disorder (ASD) is a broad neurodevelopmental disorder characterized by speech impairment, social impairment, restricted interests, and repetitive stereotypic behaviors (1). There has been an annual increase in the prevalence rate of ASD in China, reaching 1% (2). Unfortunately effective treatments strategies for ASD are lacking.

Individuals with ASD commonly present a range of gastrointestinal symptoms, as evaluated by the GSRS (3, 4). These symptoms encompass constipation, diarrhea, nausea, irregular bowel movements, abdominal discomfort, and vomiting (3). It is noteworthy that these symptoms are closely associated with the severity of autism (4). The gut microbiota is involved in the pathogenesis of ASD through the microbiota-gut-brain axis (5), thus affecting gastrointestinal function and activity (6, 7). Single-strain or multi-strain probiotics can reduce the ABC total score, CARS score, SRS score, and GRSR score in patients with ASD, thereby improving their autistic behaviors and gastrointestinal symptoms (8–23). Moreover, probiotic supplements have been used to treat patients in clinical practice. However, the effectiveness of different types of probiotic supplementation in improving core ASD symptoms remains unclear.

*Bacteroides fragilis* is proposed as second-generation probiotic (24). In 2013, Hsiao et al. (9) reported that the non-toxigenic *B. fragilis* strain NCTC9343 could improve the stereotypic and anxious behaviors in an ASD mouse model, indicating the treatment potential of *B. fragilis* for ASD. *B. fragilis* BF839 is a non-toxic intestinal symbiotic bacterium (25) that can be used to treat autoimmune diseases (26), prevent intestinal and respiratory diseases, and promote physical growth and development in children (27). The Totem Probiotic Fluid (28) is made through fermentation of *B. fragilis* and has been approved as a safe food raw material (28, 29). BF839 can improve social novelty preference and learning memory in mice with fragile X syndrome (30). Furthermore, it is clinically effective in the treatment of intractable epilepsy (31) and autoimmune-related epilepsy (32). We hypothesized that BF839 can clinically improve behavioral performance in individuals with ASD. Accordingly, we explored the therapeutic effects and safety of BF839 in the treatment of pediatric ASD.

## 2 Materials and methods

### 2.1 Participants

We included children with ASD who consulted the Second Affiliated Hospital of Guangzhou Medical University between August 2020 and September 2021. This study was conducted in accordance with the guidelines of the Declaration of Helsinki and approved by the Ethics Committee of the Second Affiliated Hospital of Guangzhou Medical University (item number 2019-hs-43). Written informed consent was obtained from all subjects involved in the study. The study was registered in Chinese Clinical Trial Registry (Clinical trial registration number ChiCTR2000035006). The inclusion criteria were as follows: (1) age 2–10 years, (2) meeting the diagnostic criteria for ASD in the fifth edition of the Diagnostic and Statistical Manual of Mental Disorders as well as the second edition of Autism Diagnostic Observation Schedule and/or Autism Diagnostic Interview-Revised, (3) >3 months of maintaining the original rehabilitation training/treatment, and (4) cooperation from the guardians in completing the corresponding assessments. The exclusion criteria were (1) comorbid psychiatric or developmental disorders; (2) taking psychotropic medications; (3) having serious cardiac, pulmonary, hepatic, renal, or hematopoietic disorders (including unstable angina, uncontrolled asthma, active gastric bleeding, and cancer); and (4) receiving antibiotics, probiotics, ketogenic diet, fecal transplantation, proton pump inhibitors, other treatment regimens with gastrointestinal effects, and other ASD treatment regimens within 1 month before enrollment. The withdrawal criteria were as follows: (1) loss of follow-up, (2) unacceptable adverse reactions or severe adverse events, (3) treatment alterations during the study period, and (4) failure to take probiotics as required (>20% underdose of the trial dosage).

### 2.2 Study methods

#### 2.2.1 Experimental design

This randomized, double-blind, placebo-controlled study was conducted following the Consolidated Standards of Reporting Trials guidelines (33). Participants were allocated (1:1) to the probiotics or placebo group using a concealed random allocation from a computer-generated random numbers table produced by Python (a cross-platform computer programming language). Based on the preliminary observations in the pre-experiment and a previous report (14) indicating a 5% reduction in the ABC total score with placebo as an adjuvant therapy, along with our pre-experiment’s reduction rate of 35%, and setting the alpha value at 0.05, power effect value at 80%, and a ratio of 1, we calculated the required sample size to be 48 (24 per group). Considering a 20% dropout rate, 60 patients (30 patients per group) were enrolled. Follow-up assessments were performed on day 0 and at weeks 8 and 16. Randomization, allocation concealment, and unblinding were performed by a blinded statistician. To ensure allocation concealment, the experimental supplies were stored in a trial product warehouse using corresponding coded boxes with the same appearance. Moreover, the same packaging was used for all treatment powders. The investigators, patients, and their guardians were double-blinded. Unblinding only occurred after the completion of the 16-week trial by all participants.

#### 2.2.2 Interventions

The BF839 group received BF839 powder (10 g/bar with ≥10^6^ CFU/bar of viable bacteria), whereas the placebo group received only maltodextrin (10 g/bar). Using the method of food sensory evaluation (34), it was determined that the products from both groups exhibited a high level of similarity in terms of both odor and taste. Patients took one strip of either product with warm water twice daily for 16 weeks. During the intervention period, all patients maintained their original educational rehabilitation training. The trial products were provided by Guangzhou Totem Life Medicine Research Co., Ltd.

#### 2.2.3 Indicators for observation, sample collection, and sequencing analysis

The primary outcomes were ABC scores. The secondary outcomes were CARS, SRS, S-M, GSRS scores, and fecal microbiome. The above outcomes were monitored on day 0, week 8, and week 16. The ABC, SRS, S-M, and GSRS were completed by parents or caregivers, while the CARS were completed by physicians.

##### 2.2.3.1 Overview of assessment tools used

###### 2.2.3.1.1 ABC

This scale (35) lists 57 behavioral characteristics of pediatric autism. It is comprised of five subscales: sensory (9 entries, 30 points), relating (12 entries, 35 points), body and object use (12 entries, 28 points), language (13 entries, 31 points), and social and self-help (11 entries, 25 points). Higher ABC scores indicate more severe behavioral ASD symptoms.

###### 2.2.3.1.2 CARS

Each CARS (35) item was rated on a 4-point scale. Further, the CARS covers 15 major domains: interpersonal relationships, imitation, affective responses, body and object use, relationships with inanimate objects, adaptation to environmental changes, visual responses, auditory responses, proximal sensory responses, anxiety responses, verbal communication, non-verbal communication, intellectual functioning, and general impressions. It has a total possible score of 60, with a higher score indicating more severe behavioral ASD symptoms.

###### 2.2.3.1.3 SRS

This scale (35) comprises 65 items, which are each rated on a 4-point scale. It is used to screen individuals aged 4–18 years for social communication and interaction as well as restricted behaviors (total score: 0–195). Specifically, it assesses the following domains: social awareness (0–24 points), social cognition (0–36 points), social communication (0–66 points), social motivation (0–33 points), and autistic mannerism (0–36 points) subscales. A higher score indicates more severe social impairment.

###### 2.2.3.1.4 S-M

This scale (35) is used to assess different life skills in children. It comprises 132 items distributed across six domains: independent living, exercise, homework, interactions, participation in group activities, and self-management. Each item is assigned one point and the total score is summed. The corresponding standard score was determined based on the age group and score range. A higher standard score indicates better social skills.

###### 2.2.3.1.5 GSRS

This scale (36) evaluates gastrointestinal symptoms and consists of 15 questions divided into 5 domains covering the gastrointestinal system: diarrhea, constipation, abdominal pain, reflux, and indigestion. The questionnaire responses are rated on a 7-point Likert scale, with “1” indicating absence and “7” representing the highest frequency or intensity of the symptoms. A higher score indicates more severe gastrointestinal symptoms.

##### 2.2.3.2 Stool sample collection

Stool was collected from 21 random patients on day 0 and week 16. We selected 9 and 12 patients from the placebo and BF839 groups, respectively; however, stool samples were not collected from one patient in each group at week 16, resulting in 17 and 23 samples, respectively. Stool samples were collected at the same time points at home using a sterile stool sample collection kit, followed by the addition of DNA preservation solution. The samples were shipped to Shenzhen 01 Life Institute Co., Ltd. at room temperature for testing. All samples were stored at −80°C before sequencing.

##### 2.2.3.3 DNA extraction from stool samples and metagenomic sequencing

DNA was extracted from 200 mg of feces using a series of chemical treatments and centrifugation steps. Further details regarding these processing are provided in Supplementary material S1. Samples with total DNA >1 ug and a brightness ratio of 1.8–2.0 were used for sequencing. A sequencing library was constructed using the NEBNext^®^ Ultra^™^ DNA Library Prep Kit; moreover, an indexed sequence was added for each sample. The generated DNA library was sequenced using an Illumina high-throughput sequencing platform. The libraries were constructed as follows: DNA was first fragmented to approximately 350 bp through ultrasound, followed by end-repair, A-tail addition, and addition of Illumina adapters through polymerase chain reaction (PCR). PCR products were purified using the AMPure XP system. To ensure the library quality, the distribution of library fragment lengths was determined using an Agilent 2100; moreover, the effective library concentration was determined through quantitative PCR.

##### 2.2.3.4 DNA quality control

A total of (40) samples from 21 patients were sequenced; moreover, raw base data of 253.32 Gbp were generated. Quality control of raw data was performed using the sequencing data quality control software (fastq_trim_filter_v5_EMBL) in Trimmomatic software, where (1) adapter sequences and sequences with >3 N bases in a single sequence were removed and (2) low-quality sequences were removed using length and quality thresholds of 30 bp and 20, respectively. The sequencing data were aligned with the human genome GRCh38 using the alignment tool SOAP2 (version 2.20); moreover, host contamination was removed with a similarity threshold of 90%. A total of 249.35 Gbp (cleaned data) were retained in the dataset for downstream analysis after the quality control step.

##### 2.2.3.5 Alignment with the reference gene set and intestinal metabolic module prediction

Gene set alignment and calculation of relative abundance were performed as previously described (37). Subsequently, gene coding-related intestinal metabolic modules were predicted using the module-based analysis method as previously described (38) and using MetaCyc, which is a metabolism database. Briefly, SOAP2 (version 2.20) was used to align the sequencing data with the reference gene set to filter the best-aligned sequences among sequences with >90% similarity as the final alignment result. The relative gene abundance was calculated as previously described (39). The relative abundances in the Kyoto Encyclopedia of Genes and Genomes Orthology (KO) database were obtained by summing and normalizing the relative abundances of the annotated genes. Contrastingly, the abundance of intestinal metabolic modules was calculated from the combinations in the KO database that comprised the intestinal metabolic modules.

##### 2.2.3.6 Species annotation

Species annotation was performed using MetaPhlAn (default parameters) and species abundance was calculated at the phylum, genus, and species levels.

##### 2.2.3.7 Bioinformatics analysis

All bioinformatics analyses were performed using R3.6.3. The observed number of species (count) and Shannon diversity of the samples were calculated using the species-level abundance file of MetaPhlAn to assess changes in the microbiota diversity in the fecal samples. The Bray–Curtis distance similarity matrix was calculated using the species-level abundance file of MetaPhlAn, followed by principal coordinates analysis to determine changes in the microbiota composition. Additionally, we performed a permutational multivariate analysis of variance analysis (999 permutation tests) using the vegan R package to determine differences in the microbiota composition according to the group and over time.

#### 2.2.4 Indicators for safety

Adverse events related to the treatment, including nausea, vomiting, diarrhea, constipation, increased flatulence, and rash, were recorded, and monitored on day 0, week 8, and week 16.

#### 2.2.5 Statistical methods

Efficacy was assessed in the full analysis set, which included all randomized patients who received at least one post-intervention assessment. SPSS version 22.0 (SPSS, Inc., Chicago, IL, United States) was used for all statistical analyses. Normally and non-normally distributed measurement data are expressed as mean ± standard deviation ( $\bar{x}$ ± *s*) and median (quartile) [*M*(*Q*1, *Q*3)] values, respectively; the *t*-test and Mann–Whitney *U* test, were used for between groups comparisons, respectively; the paired *t*-test and Wilcoxon signed-rank test, were used for within group comparisons, respectively. Correlation analysis was performed using Pearson’s correlation coefficient. Count data are expressed as the number of cases (%); the chi-square test or Fisher’s exact test were used for between-group comparisons. Statistical significance was set at *p* < 0.05.

## 3 Results

### 3.1 Baseline characteristics

Of 98 patients screened between August 31, 2020, and September 10, 2021, we included 60 patients (53 males and 7 females; median age, 4.23 (3.21, 5.17) years; age range, 2.30–10.00 years; placebo group: *n* = 30, BF839 group: *n* = 30). There were no significant between-group differences (*p* > 0.05) in demographic indicators, including age, sex, disease duration, weight, and height, as well as in the clinical indicators, including the ABC, CARS, SRS, S-M, and GSRS scores. The baseline characteristics of the patients are shown in Table 1.

**Table 1:** Baseline demographic and clinical characteristics of children with ASD in both groups (*n* = 60 cases, randomized set).

Characteristic	Placebo group (*n* = 30)	BF839 group (*n* = 30)
Age [year, *M*(*Q*_1_, *Q*_3_)]	4.20 (3.50, 5.33)	4.20 (3.00, 5.17)
Sex (case, %)		
Female	3 (10.00)	4 (13.33)
Male	27 (90.00)	26 (86.67)
Period of disease [year, *M*(*Q*_1_, *Q*_3_)]	1.70 (1.00, 2.00)	1.50 (1.00, 3.00)
Weight [kg, *M*(*Q*_1_, *Q*_3_)]	16.00 (14.00, 20.50)	16.50 (13.88, 19.00)
Height (cm, $\bar{x}$ ± *s*)	105.35 ± 10.26	105.77 ± 11.61
ABC score ( $\bar{x}$ ± *s*)	52.13 ± 22.88	53.33 ± 22.40
CARS score ( $\bar{x}$ ± *s*)	32.00 ± 8.40	32.35 ± 7.51
SRS score ( $\bar{x}$ ± *s*)^a^	102.17 ± 24.81	104.56 ± 23.17
S-M standardized score [*M*(*Q*_1_, *Q*_3_)]	9.00 (8.00, 9.50)	8.50 (8.00, 9.00)
GSRS score [*M*(*Q*_1_, *Q*_3_)]	23.00 (19.50, 25.50)	21.50 (19.00, 26.75)

a The SRS scale only evaluated children aged >4 years (Placebo group: *n* = 18; BF839 group: *n* = 16).

**Figure 1:**
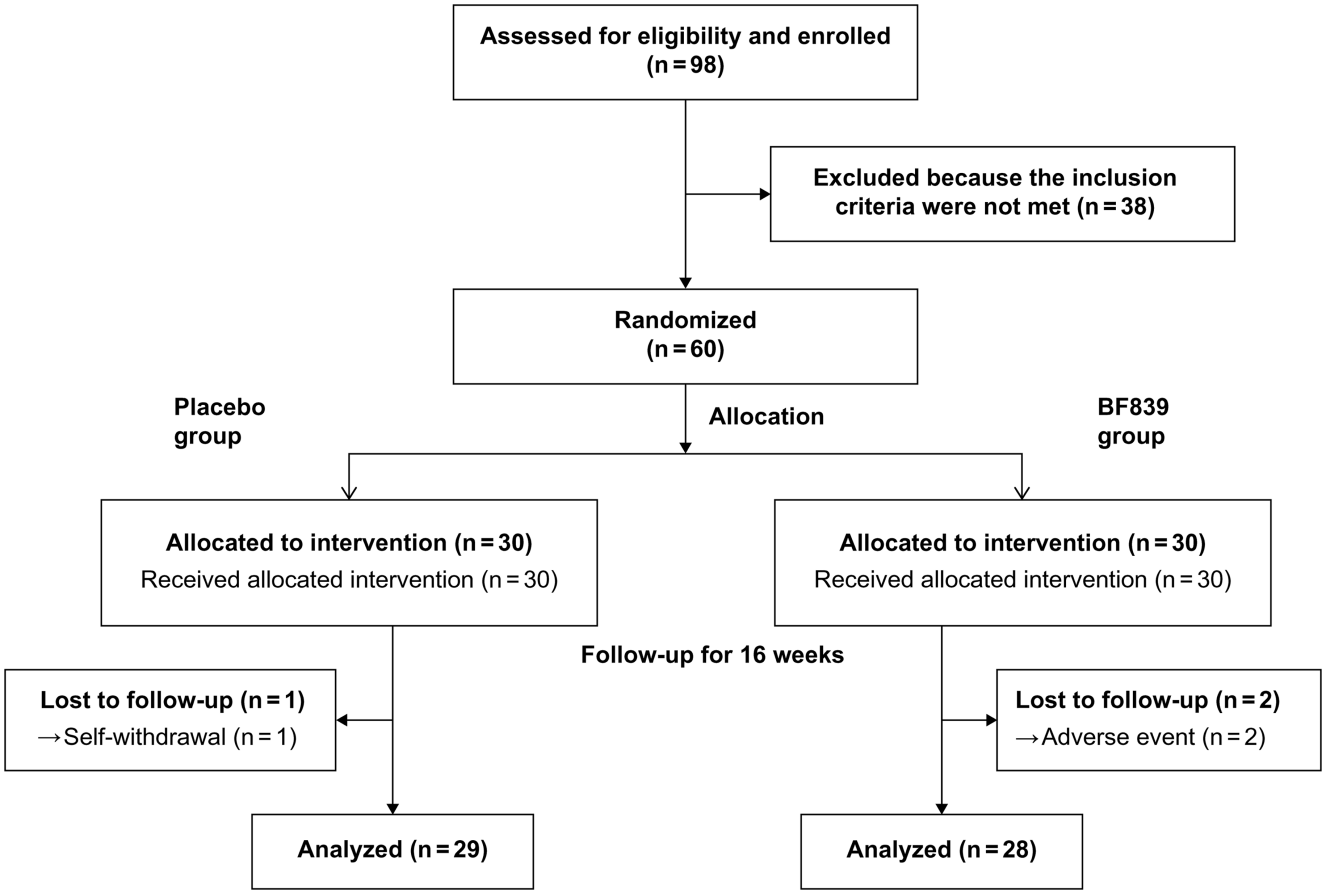
Trial flowchart.

Three patients dropped out during the study period. One patient in the placebo group withdrew due to failure to return on time for the 8-week follow-up due to COVID-19 restrictions. Two patients withdrew from the BF839 group after the parents withdrew consent due to adverse events of mild diarrhea after 4 weeks. Finally, 57 patients completed the experiment (Figure 1), 29 and 28 in the placebo and BF839 groups, respectively. No other adverse events were reported.

### 3.2 Correlation between clinical and gastrointestinal symptoms

We observed that the total ABC, sensory, relating, body and object use, language, social, and self-help scores were significantly positively correlated with the GSRS score (*p* < 0.05) (Supplementary Table S1).

### 3.3 Between-group comparison of score improvements after 8 and 16 weeks

At week 16, the BF839 group showed significantly improved ABC body and object use score compared with the placebo group (−4.68 ± 6.29 vs. −1.07 ± 5.73, *p* = 0.026). Additionally, the BF839 group showed non-significantly better improvement in the ABC total score (−15.43 ± 18.62 vs. −9.40 ± 17.09), SRS score (−13.89 ± 17.95 vs. −12.28 ± 17.10), standard S-M score [1.00 (0.00, 1.00) vs. 0.00 (0.00, 0.00]), and GSRS score [−3.00 (−4.75, 2.75) vs. 0.00 (−3.50, 3.00)] than did the placebo group (*p* > 0.05). At week 8, the placebo group demonstrated a significant improvement in the ABC total score and ABC relating score compared to baseline (*p* < 0.05). Furthermore, after 16 weeks of intervention, both the BF839 group and placebo group showed significant improvements compared to baseline (*p* < 0.05). The BF839 group exhibited enhancements in the ABC total score, ABC subscale scores (excluding sensory score), CARS score, SRS score, and SRS Awareness score. Meanwhile, the placebo group also demonstrated improvements in the ABC total score, ABC subscale scores (excluding body and object use score and social and self-help score), CARS score, SRS score, SRS cognition score, and SRS communication score. Table 2 summarizes the results.

**Table 2:** Between-group comparison of the scores of children with ASD after 8 and 16 weeks of intervention [ $\bar{x}$ ± *s*/*M*(*Q*1, *Q*3), full analysis set].

Scale	Baseline (0 day)		Difference from baseline after 8 weeks of treatment			Difference from baseline after 16 weeks of treatment		
	Placebo group	BF839 group	Placebo group	BF839 group	*p*-value	Placebo group	BF839 group	*p*-value
Number of cases	30	30	29	28		29	28	
ABC total score	52.13 ± 22.88	53.33 ± 22.40	−6.00 ± 11.24^b^	−6.39 ± 24.01	0.937	−9.40 ± 17.09^b^	−15.43 ± 18.62^b^	0.204
Sensory	9.57 ± 5.73	9.83 ± 4.78	0.38 ± 4.34	0.18 ± 7.05	0.897	−0.03 ± 4.50^b^	−0.68 ± 7.11	0.679
Relating	11.53 ± 5.74	10.73 ± 5.91	−1.66 ± 4.12^b^	−0.71 ± 5.61	0.472	−2.00 ± 4.56^b^	−1.57 ± 6.06^b^	0.761
Body and object use	5.97 ± 6.23	8.87 ± 8.59	−0.55 ± 3.10	−0.89 ± 8.51	0.840	−1.07 ± 5.73	−4.68 ± 6.29^b^	0.026^a^
Language	14.37 ± 6.69	13.63 ± 6.06	−1.76 ± 5.41	−1.79 ± 7.89	0.988	−2.93 ± 6.65^b^	−3.86 ± 6.04^b^	0.583
Social and self-help	10.70 ± 5.04	10.63 ± 5.95	−0.93 ± 5.68	−1.04 ± 5.36	0.943	−1.43 ± 5.16	−2.58 ± 4.55^b^	0.378
Number of cases	30	30	29	28		29	28	
CARS score	32.00 ± 8.40	32.35 ± 7.51	−1.03 ± 3.25	−1.27 ± 4.01	0.810	−3.00 ± 4.97^b^	−4.31 ± 5.31^b^	0.338
Number of cases	18	16	17	14		18	15	
SRS score^c^	99.90 ± 24.21	99.41 ± 22.81	−4.39 ± 14.70	−5.04 ± 18.50	0.887	−12.28 ± 17.10^b^	−13.89 ± 17.95^b^	0.732
Awareness	12.86 ± 3.24	12.21 ± 2.61	−0.46 ± 2.47	−0.15 ± 2.60	0.655	−1.62 ± 3.48	−1.48 ± 2.81^b^	0.870
Cognition	20.31 ± 4.78	19.41 ± 4.94	−0.57 ± 3.46	−0.54 ± 5.10	0.978	−2.45 ± 4.36^b^	−2.11 ± 2.91	0.737
Communication	36.27 ± 9.60	34.55 ± 8.84	−2.29 ± 6.02	−1.04 ± 6.94	0.483	−4.48 ± 6.61^b^	−4.41 ± 7.38	0.968
Motivation	15.67 ± 4.05	16.38 ± 5.14	−0.18 ± 3.91	−1.62 ± 4.19	0.198	−1.17 ± 4.05	−2.30 ± 5.19	0.369
Mannerisms	14.79 ± 6.86	16.86 ± 6.84	−0.89 ± 4.13	−1.69 ± 5.00	0.524	−2.55 ± 5.10	−3.59 ± 4.49	0.422
Number of cases	30	30	29	28		29	28	
S-M standardized score	9.00 (8.00, 9.50)	8.50 (8.00, 9.00)	0.00 (0.00, 0.00)	1.00 (0.00, 1.00)	0.268	0.00 (0.00, 0.00)	1.00 (0.00, 1.00)	0.154
Number of cases	30	30	29	28		29	28	
GSRS score	23.00 (19.50, 25.50)	21.50 (19.00, 26.75)	−1.00 (−3.00, 3.00)	−2.00 (−4.75, 3.75)	0.767	0.00 (−3.50, 3.00)	−3.00 (−4.75, 2.75)	0.138

a *p < 0.05 compared with the placebo group.*

b *p < 0.05 compared with the baseline.*

c At the time of the SRS assessment, one child over 4 years of age from the BF839 group dropped out, and one participant in the placebo group and one in the BF839 group missed completing the questionnaire at week 8.

### 3.4 Between-group comparison of score improvements according to age

We performed further subgroup analysis according to age (<4 years and ≥ 4 years old). Among children aged <4 years, the BF839 group showed significant improvement in the ABC body and object use score after 16 weeks, compared with the placebo group (−4.85 ± 4.60 vs. 1.50 ± 3.87, *p* = 0.001). Furthermore, the ABC total score and each of the ABC subscale scores, CARS score, standard S-M score, and GSRS score at week 16 showed non-significantly better improvement than did the placebo group (*p* > 0.05). A similar trend was observed at week 8; however, it was not as pronounced as at week 16. At week 8, only the BF839 group showed significantly better improvement (*p* < 0.05) in ABC sensory score than the baseline. At week 16, compared to baseline, the BF839 group exhibited significant improvements in the ABC total score, ABC subscale scores (excluding relating and social and self-help), CARS score, and standard S-M score (*p* < 0.05). In contrast, only the ABC sensory score and CARS score showed significant improvement (*p* < 0.05) in the placebo group. Table 3 summarizes the results.

**Table 3:** Comparison of the improvement in the scores of children with ASD of <4 years old between the two groups after 8 and 16 weeks of intervention [ $\bar{x}$ ± *s*/*M*(*Q*1, *Q*3), full analysis set].

Scale	Baseline (0 day)		Difference from baseline after 8 weeks of treatment			Difference from baseline after 16 weeks of treatment		
	Placebo group (*n* = 12)	BF839 group (*n* = 14)	Placebo group (*n* = 12)	BF839 group (*n* = 13)	*p*-value	Placebo group (*n* = 12)	BF839 group (*n* = 13)	*p*-value
ABC total score	51.42 ± 5.55	54.50 ± 7.23	−1.17 ± 14.29	−12.15 ± 28.79	0.245	−6.42 ± 15.96	−20.23 ± 23.92^b^	0.106
Sensory	10.50 ± 1.61	10.29 ± 1.53	0.58 ± 4.78	−1.85 ± 6.12^b^	0.283	−0.25 ± 4.88^b^	−2.08 ± 6.63^b^	0.444
Relating	11.83 ± 1.82	11.00 ± 1.80	−0.75 ± 2.67	−2.62 ± 6.20	0.346	−1.58 ± 2.84	−2.85 ± 7.46	0.588
Body and object use	4.42 ± 1.35	9.36 ± 1.96	−0.33 ± 2.77	−0.92 ± 10.85	0.857	1.50 ± 3.87	−4.85 ± 4.60^b^	0.001^a^
Language	13.92 ± 2.07	14.36 ± 1.86	−0.42 ± 6.37	−2.92 ± 9.16	0.439	−2.83 ± 7.51	−5.31 ± 7.35^b^	0.415
Social and self-help	10.75 ± 1.16	10.21 ± 1.66	1.75 ± 6.74	−1.38 ± 4.48	0.181	−0.083 ± 5.65	−3.00 ± 5.93	0.221
CARS score	33.33 ± 2.31	32.07 ± 2.11	−0.67 ± 2.67	−1.38 ± 4.81	0.646	−2.67 ± 2.99^b^	−5.23 ± 6.47^b^	0.223
S-M standardized score	8.00 (8.00, 9.75)	9.00 (8.75, 9.25)	0.00 (0.00, 0.75)	0.00 (0.00, 1.00)	0.485	0.00 (0.00, 1.00)	1.00 (0.00, 1.00)^b^	0.585
GSRS score	22.50 (19.25, 24.00)	21.50 (18.75, 28.00)	−1.50 (−6.00, 3.75)	−2.00 (−6.00, 2.50)	0.849	0.00 (−4.00, 3.00)	−2.00 (−5.50, 1.00)	0.155

a *p < 0.05 compared with the placebo group.*

b *p < 0.05 compared with the baseline.*

Among children aged ≥4 years, there was no between-group difference in the improvement after 8 and 16 weeks of intervention. At week 8, only the placebo group demonstrated a significant improvement in the ABC total score, ABC language score, and ABC social and self help compared to baseline (*p* < 0.05). Nevertheless, after 16 weeks, the BF839 group showed significant differences (*p* < 0.05) in ABC total score and ABC subscale scores (excluding sensory score and relating score), CARS score, and S-M standardized score compared with baseline, while the placebo group only had significant improvement (*p* < 0.05) in ABC total score and CARS score. The results are summarized in Supplementary Table S2.

### 3.5 Between-group comparison of the score improvements according to baseline disease severity

We performed further subgroup analysis according to baseline disease severity (CARS score: <30 and ≥30). Among children with a baseline CARS score ≥30, the BF839 group demonstrated significant improvements in the ABC total score (−19.71 ± 24.12 vs. −5.05 ± 16.58, *p* = 0.047), ABC body and object use score (−5.71 ± 8.26 vs. −0.32 ± 5.88, *p* = 0.034), and CARS score (−5.57 ± 5.79 vs. −2.11 ± 3.70, *p* = 0.044) compared with the placebo group. Moreover, the BF839 group demonstrated significant improvement in the GSRS score at both week 8 [−3.50 (−7.36, −1.25) vs. 0.00 (−3.00, 3.00), *p* = 0.045] and week 16 [−3.50 (− 7.13, −1.25) vs. 2.00 (−3.00, 3.00), *p* = 0.014]. At week 8, both the placebo group and the BF839 group showed significantly better improvement in the S-M standardized score than the baseline (*p* < 0.05). At week 16, compared to baseline, the BF839 group exhibited significant improvements (*p* < 0.05) in the ABC total score, ABC language score, CARS score, standard S-M score, and GSRS score. In contrast, only the CARS score showed significant improvement (*p* < 0.05) in the placebo group. The results are summarized in Table 4.

**Table 4:** Comparison of the improvement in the scores of children with ASD of a baseline CARS score of ≥30 between the two groups after 8 and 16 weeks of intervention [ $\bar{x}$ ± *s*/*M*(Q1, Q3), full analysis set].

Scale	Baseline (0 day)		Difference from baseline after 8 weeks of treatment			Difference from baseline after 16 weeks of treatment		
	Placebo group (*n* = 19)	BF839 group (*n* = 15)	Placebo group (*n* = 19)	BF839 group (*n* = 14)	*p*-value	Placebo group (*n* = 19)	BF839 group (*n* = 14)	*p*-value
ABC total score	58.63 ± 4.33	65.27 ± 5.78	−3.73 ± 12.80	−5.21 ± 29.00	0.861	−5.05 ± 16.58	−19.71 ± 24.12^b^	0.047^a^
Sensory	11.05 ± 1.01	12.00 ± 1.27	0.26 ± 4.09	1.00 ± 7.20	0.712	0.21 ± 4.26	−1.29 ± 8.20	0.500
Relating	12.47 ± 1.24	14.33 ± 1.46	−1.16 ± 4.31	−1.50 ± 5.89	0.848	−1.21 ± 4.22	−3.79 ± 6.96	0.234
Body and object use	7.42 ± 1.39	12.13 ± 2.63	−0.53 ± 3.08	−0.07 ± 11.15	0.884	−0.32 ± 5.88	−5.71 ± 8.26	0.034^a^
Language	15.58 ± 1.30	15.87 ± 1.54	−0.32 ± 5.88	−2.29 ± 9.93	0.516	−0.84 ± 6.75	−4.57 ± 7.03^b^	0.134
Social and self-help	12.10 ± 1.09	11.67 ± 1.77	−0.21 ± 6.47	0.00 ± 5.19	0.921	−0.47 ± 5.74	−3.14 ± 5.70	0.195
CARS score	35.58 ± 0.98	38.40 ± 1.39	−1.16 ± 3.37	−1.86 ± 4.20	0.600	−2.11 ± 3.70^b^	−5.57 ± 5.79^b^	0.044^a^
S-M standardized score	8.00 (8.00, 9.00)	8.00 (7.00, 9.00)	0.00 (0.00, 0.00)^b^	0.00 (−0.25, 1.00)^b^	0.702	0.00 (0.00, 1.00)	0.50 (0.00, 1.00)^b^	0.393
GSRS score	23.00 (20.00, 24.00)	22.00 (20.00, 31.00)	0.00 (−3.00, 3.00)	−3.50 (−7.36, −1.25)	0.045^a^	2.00 (−3.00, 3.00)	−3.50 (−7.13, −1.25)^b^	0.014^a^

a *p < 0.05 compared with the placebo group.*

b *p < 0.05 compared with the baseline.*

Among children with a baseline CARS score <30, there was no significant between-group difference in the improvement (*p* > 0.05) after 8 and 16 weeks of intervention. At week 8, compared to the baseline, the BF839 group showed significant improvements (*p* < 0.05) in the ABC sensory score and S-M standardized score, while the placebo group showed significant improvements (*p* < 0.05) in the ABC total score and ABC language score. After 16 weeks of intervention, compared to the baseline, both the BF839 group and placebo group demonstrated significant improvements (*p* < 0.05). The BF839 group showed enhancements in the ABC total score, ABC subscale scores (excluding relating score), S-M standardized score, and CARS score. Meanwhile, the placebo group exhibited improvements in the ABC total score, ABC language score, ABC social and self-help score, as well as CARS score. The results are summarized in Supplementary Table S3.

### 3.6 Changes in the intestinal microbiota

**Figure 2:**
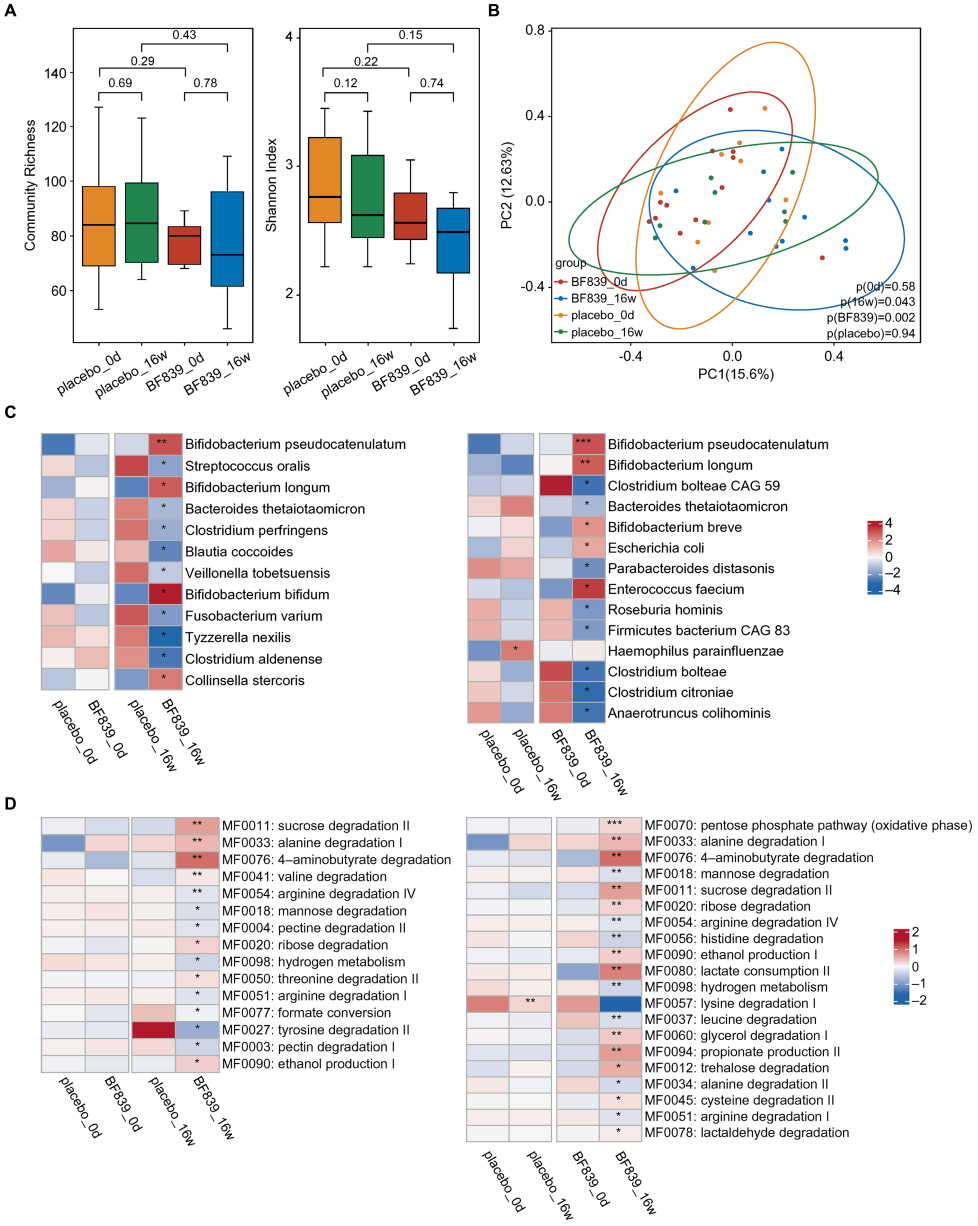
Changes in the intestinal microbiota. **(A)** The diversity of fecal microbiota at the species level between the placebo group and the BF839 group on day 0 and at week 16. Left: community abundance. Right: Shannon diversity index. Elements of box plot: centerline: median; ends of the box: upper and lower quartiles; dot: outlier. **(B)** PCoA scores for the placebo and BF839 groups on day 0 and at week 16 with different colors for each group of samples. **(C)** Between-group differences in microbiota at the species level. Left: between-group comparison of abundance on day 0 and at week 16, respectively. There were significant between-group differences in the microbial abundance at week 16 but not on day 0. Right: within-group comparison of the abundance between day 0 and at week 16. There were significant increases/decreases in abundance in both groups at week 16 compared with that on day 0. ^*^p < 0.05 and ^**^p < 0.01. **(D)** Analysis of the intestinal metabolic modules (GMMs). Left: between-group comparison of the distribution of intestinal metabolic modules on day 0 and at week 16, respectively. There were significant between-group differences at week 16, but not on day 0. Right: within-group comparison of the distribution of intestinal metabolic modules between day 0 and at week 16. There were significant increases/decreases in abundance in both groups at week 16 compared with that on day 0. ^*^p < 0.05 and ^**^p < 0.01.

There was no significant between-group difference in the changes in microbiota diversity at week 16 (Figure 2A). Further, there were no significant microbiota changes between day 0 and week 16 (Figure 2A). Contrastingly, in the BF839 group, there was a non-significant decrease in the microbiota diversity at week 16 compared with that on day 0 (Figure 2A). Additionally, principal coordinates analysis (PCoA) revealed no significant between-group difference in the microbiota composition on day 0 (*p* = 0.58), as well as between day 0 and week 16 in the placebo group (*p* = 0.94) (Figure 2B). However, there was a significant between-group difference in the microbiota composition at week 16 (*p* = 0.043), as well as between day 0 and week 16 in the BF839 group (*p* = 0.002) (Figure 2B).

A finer taxonomic analysis revealed regulation of the fecal microbiota driven by the intervention process at the species level. There was no significant between-group difference in the abundance on day 0. However, the BF839 group at week 16 demonstrated an increase in the abundance of *Bifidobacterium pseudocatenulatum*, *Bifidobacterium longum*, *Bifidobacterium bifidum*, and *Collinsella stercoris*, as well as a decrease in the abundance of *Streptococcus oralis*, *Bacteroides thetaiotaomicron*, *Clostridium perfringens*, *Blautia coccoides*, *Veillonella tobetsuensis*, *Fusobacterium varium*, *Tyzzerella nexilis*, and *Clostridium aldenense* compared with the placebo group at week 16 (Figure 2C). Within-group comparisons indicated that the placebo group showed greater abundance of *Haemophilus parainfluenzae* at week 16 than on day 0. Moreover, the BF839 group showed greater abundance of *Bifidobacterium pseudocatenulatum*, *Bifidobacterium longum*, *Bifidobacterium breve*, *Escherichia coli*, and *Enterococcus faecium* and a decreased abundance of *Clostridium bolteae* CAG 59, *Bacteroides thetaiotaomicron*, *Parabacteroides distasonis*, *Roseburia hominis*, *Firmicutes bacterium* CAG 83, *Clostridium bolteae*, *Clostridium citroniae*, and *Anaerotruncus colihominis* at week 16 than on day 0 (Figure 2C).

We conducted a comprehensive analysis of intestinal metabolic function modules. The findings were based on microbial genome analysis reflecting potential metabolic capacity within the flora. We observed no significant between-group differences in the metabolic functions of neuroactive compounds encoded by intestinal microorganisms on day 0. However, the BF839 group at week 16 showed significant increases in several metabolic functions, including sucrose degradation II, alanine degradation I, 4-aminobutyrate degradation, valine degradation, ribose degradation, threonine degradation II, and ethanol production I, as well as significant decreases in several metabolic functions, including arginine degradation IV, mannose degradation, pectin degradation II, hydrogen metabolism, arginine degradation I, formate conversion, tyrosine degradation II, and pectin degradation I, compared with the placebo group at week 16 (Figure 2D). Within-group comparison indicated that the placebo group at week 16 demonstrated a decrease in lysine degradation I compared with that on day 0; contrastingly, the BF839 group at week 16 demonstrated significant increases in the pentose phosphate pathway (oxidative phase), alanine degradation I, 4-aminobutyrate degradation, sucrose degradation II, ribose degradation, ethanol production I, lactate consumption II, glycerol degradation I, propionate production II, trehalose degradation, cysteine degradation II, and lactaldehyde degradation, as well as significant decreases in mannose degradation, arginine degradation IV, histidine degradation, hydrogen metabolism, leucine degradation, alanine degradation II, and arginine degradation I compared with those on day 0 (Figure 2D).

## 4 Discussion

We observed a positive correlation between the ABC total score and the GSRS score as well as between each of the ABC subscale scores and the GSRS score, which is consistent with previous reports (21–23). This demonstrates that abnormal autistic behaviors are more pronounced with increasing severity of gastrointestinal symptoms such as diarrhea, constipation, abdominal pain, reflux, and indigestion, and thus improving gastrointestinal symptoms can attenuate autistic symptoms.

After 16 weeks of intervention, both the placebo group and the BF839 group exhibited significant decreases in ABC total score, SRS score, and CARS score compared to the baseline, indicating significant improvements in abnormal behavior and social interaction of ASD. It is noteworthy that both groups received rehabilitation training, a widely recognized method in autism treatment supported by existing studies (40, 41) for its ability to improve ASD behavior. Moreover, as the patients aged during the intervention process, natural improvements in the ability of language and social and self-help may have occurred due to brain maturation and accumulated life experiences (42, 43). However, the developmental ability level remains below that of normal children. Therefore, the ASD behavior in the placebo group can be improved, which is related to the above reasons.

Notably, the BF839 group showed significant improvement in the ABC body and object use scores compared with the placebo group at week 16, which was more pronounced in children with ASD aged <4 years. Among children with a baseline CARS score of ≥30, the BF839 group showed significant improvements at week 16 in the ABC total score, ABC body and object use score, CARS score, and GSRS score compared with the placebo group. To our knowledge, this is the first randomized, double-blind, placebo-controlled clinical trial to show that probiotics can significantly improve overall ASD symptoms, especially body and object use, which reflects stereotypical behaviors.

Compared with the placebo group, the BF839 group showed significant improvement at week 16 in the ABC body and object use score among children aged <4 years, but not those aged ≥4, years. Moreover, the BF839 group showed greater improvement in all scale scores in children aged <4 years than in children aged ≥4 years. These results reaffirm the importance of early intervention in children with ASD (44) and suggest that an age of <4 years may be a window of effectiveness for probiotic intervention.

Liu et al. (13) reported no significant difference in the ABC1 total and SRS scores between the placebo and *Lactobacillus plantarum* 128 (PS128) groups after 4 weeks of intervention in patients with ASD. Similarly, Kong et al. (14) reported no significant difference in the ABC total score, ABC subitem scores, SRS total score, and SRS subitem scores between the PS128 and placebo groups after 16 weeks of intervention in patients with ASD. Arnold et al. (18) observed no significant differences in the scores for stereotype and inappropriate speech in ABC2 and SRS between the probiotic and placebo groups after 19 weeks of intervention, with the probiotics comprising multiple strains, including four strains of *Lactobacilli* (*Lactobacillus casei*, *Lactobacillus plantarum*, *Lactobacillus acidophilus*, and *Lactobacillus delbrueckii* subspecies *bulgaricus*), three strains of *bifidobacteria* (*Bifidobacterium longum*, *Bifidobacterium infantis* and *Bifidobacterium breve*), one strain of *Streptococcus thermophiles*, and starch. Contrastingly, our findings suggest that BF839 has excellent efficacy in improving behavioral symptoms in children with ASD.

Zhao et al. (20) reported a 10.8% reduction in the CARS score at 2 months after enteroscopic and gastroscopic fecal microbiota transplantation (FMT) (only a 0.8% reduction in the control group, *p* < 0.001). In our study, among children with a baseline CARS score of ≥30, it reduced by 14.50% (5.57/38.40) and 5.93% (2.11/35.58) in the BF839 and placebo groups, respectively (*p* = 0.044) (Table 4), which is consistent with the efficacy of FMT. However, the rate of adverse events in the Zhao’s (20) and our current study 29.2% and 6.67% (2/30), demonstrating the superiority of BF839 in terms of safety and operability.

Regarding gastrointestinal symptoms, among children with a baseline CARS score of ≥30, the BF839 group showed significant improvement in the GSRS score at both 8 and 16 weeks of intervention compared with the placebo group. This suggests that BF839 significantly improves the gastrointestinal function in children with ASD, which is consistent with previous reports (45).

Notably, there was no between-group difference in the improvement of all scale scores among children with a baseline CARS score of <30, which could be attributed to children with borderline ASD requiring a longer therapeutic duration given the less room for short-term improvement or insufficient sensitivity of the scale assessment.

*B. fragilis* and its capsular polysaccharide-A (PSA) are the most explored single commensal microbiota/symbiotic factor (46). The non-toxigenic *B. fragilis* exerts profound physiological effects (47), including prevention of intestinal inflammation in animal models of colitis (48–50) prevention of experimental autoimmune encephalomyelitis (51, 52), and activation of intestinal sensory neurons (53). Hsiao et al. (9) suggested that *B. fragilis* NCTC9343 could mitigate anxiety-like behaviors in the offspring of the mice with MIA by altering the metabolism of 4-ethyl phenyl sulfate/ester (4-EPS) and indolyl-3-acryloylglycine. These metabolites can influence the serum levels of 5-hydroxytryptamine (5-HT), and thus affect hippocampal learning and memory in the hippocampus. Multiple mechanisms related to intestinal microbiota function in ASD have been proposed, including immune activation/dysfunction, bacterial toxins (such as endotoxins, phenols, p-cresol, and 4-EPS), fermentation changes in metabolites or products (such as propionic acid and other short-chain fatty acids), and dysregulated metabolism of free amino acids (54).

Compared with healthy children, children with ASD have significantly decreased *Bifidobacterium* spp. (55–57) and *Veillonella* spp. (58). Some *Bifidobacterium* spp. produce-aminobutyric acid (GABA) (59); therefore, children with ASD present lower GABA levels. GABA is closely related to the metabolism of glutamate, which is the main excitatory neurotransmitter in the brain (60). Decreased glutamate levels are correlated with the severity of anxiety, social impairment, and behavioral impairment, which are typical symptoms of ASD (61, 62). This suggested that GABA/glutamate anomalies may be crucially involved in the pathology of ASD (61, 63). Additionally, amino acid dysregulation has been reported in children with autism (64), with intestinal microbiota being involved in amino acid metabolism. Furthermore, *Bifidobacterium longum* NCC3001 can normalize anxiety-like behaviors and hippocampal brain-derived neurotrophic factor levels in mice with infectious colitis via the vagal nerve (65). Contrastingly, we observed an increased abundance of *Bifidobacterium pseudocatenulatum*, *Bifidobacterium longum*, and *Bifidobacterium bifidum* in the BF839 group compared with that in the placebo group and the abundance at baseline. Our findings suggest that BF839 can promote intestinal growth of *Bifidobacterium* spp. in children with ASD, which significantly alters the metabolism of some neuroactive compounds. This may be among the factors underlying the observed effectiveness of BF839 in children with ASD. Nonetheless, the detailed mechanisms underlying this efficacy remain unclear.

Studies have indicated that abnormalities in the sucrose and ribosome metabolic pathways (66), branched chain amino acid catabolic function (67), and pentose phosphate metabolic pathway (68) are closely associated with the development of ASD. The sucrose metabolic pathway has been found to have a protective effect against neurodegenerative diseases (69), while ribosomal proteins may play a crucial role in maintaining cognitive function (70). Increased catabolic pathways of branched chain amino acids, such as reduced leucine content, have been linked to autism (67). Additionally, the pentose phosphate pathway is impaired in mice with autism (68). In the aforementioned metabolic pathways, it was observed that compared to day 0 and placebo groups, the BF839 group showed a significant increase in the metabolic functions of sucrose degradation II, valine degradation, ribose degradation metabolism, and pentose phosphate pathway after 16 weeks of intervention. Meanwhile, the metabolic function of leucine degradation was significantly reduced. This potentially explains the effectiveness of BF839 in treating ASD in children. However, the specific mechanism behind this effect remains unclear. Furthermore, the above findings are based on microbial genome analysis reflecting potential metabolic capacity within the flora. The exact changes of neuroactive compounds in ASD patients still requires further metabonomic analysis for confirmation.

Our findings have important practical and theoretical implications in terms of the use of probiotics in the treatment of neurodevelopmental disorders. The results of this study may provide a new, safe and effective treatment strategy for ASD. However, one limitation of this study was the short duration of the intervention, given the importance of the long-term prognosis of patients with ASD. Further cohort studies are warranted to observe the long-term effects and safety of BF839 in children with ASD.

## Data Availability

The original contributions presented in the study are included in the article/Supplementary material, further inquiries can be directed to the corresponding author.
