# Supplementary material for: Efficacy and safety of Bacteroides fragilis BF839 for pediatric autism spectrum disorder: a randomized clinical trial
Source: Front Nutr. 2024 Sep 3;11:1447059. doi: 10.3389/fnut.2024.1447059 (PMC11407114; doi:10.3389/fnut.2024.1447059)
Supplement: Supplementary file 1 [file Table_1.docx]

Supplementary Material

# Supplementary Methods

## Method S1: DNA extraction from stool samples

A stool sample of 200 mg was mixed well with ethylenediaminetetraacetic acid (0.5 M, pH 8.0) and 550 uL of lysis buffer, followed by the addition of Proteinase K and 20 μL of lysozyme and incubation at 55 °C for 2 h. Subsequently, the mixture was centrifuged at 12000 rpm for 5 min, followed by supernatant aspiration, adding 5 M of sodium chloride (NaCl), gently vortexing for 10 s, allowing to stand at -20 °C for several minutes, and re-centrifuging at 12000 rpm for 10 min. The supernatant was aspirated into an Eppendorf (EP) tube, followed by centrifugation at 12000 rpm for 5 min, supernatant aspiration into another new EP tube, isopropanol addition, allowing to stand at -20 °C for 20 min, and re-centrifugation at 12000 rpm for 10 min. Next, the liquid was added without removing the precipitate. Subsequently, the precipitate was washed twice using 1 mL 75% ethanol, with the collection of the remaining liquid by centrifugation and aspiration using a pipette tip. To avoid over-drying the DNA sample, the EP tube was placed under a clean bench with the cap open for blow-drying. Finally, 50 uL of double-distilled water (ddH2O) was added to dissolve the DNA sample, followed by the addition of 1 uL of ribonuclease (RNase) A, sufficient mixing, and incubation at 37 °C for 15 min.The degree of DNA degradation/contamination and the total DNA amount were assessed by Qubit 2.0 using a 1% agarose gel.

# Supplementary Tables

## Table S1. Analysis of the correlations between the clinical and gastrointestinal symptoms in ASD (n=57 cases, full analysis set)

|  | | **ABC Total Score** | **Sensory** | **Relating** | **Body and object use** | **Language** | **Social and self help** |
| --- | --- | --- | --- | --- | --- | --- | --- |
| **GSRS** | **Pearson R^2^** | 0.457 | 0.394 | 0.423 | 0.266 | 0.270 | 0.322 |
|  | ***P* value** | <0.001 | 0.002 | 0.001 | 0.046 | 0.042 | 0.015 |

## Table S2. Comparison of the improvement in the scores of children with ASD who were ≥4 years old between the two groups after 8 and 16 weeks of intervention [$\bar{x}$±*s*/M(Q1,Q3), full analysis set]

| **Scale** | **Baseline（0d）** | | **Difference from baseline after 8 weeks of treatment** | | | **Difference from baseline after 16 weeks of treatment** | | |
| --- | --- | --- | --- | --- | --- | --- | --- | --- |
|  | **Placebo group (n=18)** | **BF839 group(n=16)** | **Placebo group(n=17)** | **BF839 group(n=15)** | ***P* value** | **Placebo group (n=17)** | **BF839 group(n=15)** | ***P* value** |
| ABC Total Score | 52.61 ± 6.03 | 52.31 ± 4.56 | -9.41 ± 7.12^b^ | -1.40 ± 18.55 | 0.133 | -8.88 ± 14.93^b^ | -11.27 ± 11.74^b^ | 0.623 |
| Sensory | 8.94 ± 1.39 | 9.44 ± 0.98 | 0.24 ± 4.15 | 1.93 ± 7.52 | 0.446 | 0.76 ± 3.47 | 0.53 ± 7.51 | 0.914 |
| Relating | 11.33 ± 1.30 | 10.50 ± 1.33 | -2.29 ± 4.87 | 0.93 ± 4.63 | 0.065 | -1.76 ± 5.18 | -0.47 ± 4.52 | 0.459 |
| Body and object use | 7.00 ± 1.66 | 8.44 ± 2.44 | -0.71 ± 3.37 | -0.87 ± 6.23 | 0.927 | -1.94 ± 5.25 | -4.53 ± 7.62^b^ | 0.267 |
| Language | 14.67 ± 1.54 | 13.00 ± 1.33 | -2.71 ± 4.58^b^ | -0.80 ± 6.76 | 0.366 | -2.12 ± 5.15 | -2.60 ± 4.50^b^ | 0.781 |
| Social and self help | 10.67 ± 1.35 | 11.00 ± 1.47 | -2.82 ± 4.00^b^ | -0.73 ± 6.16 | 0.259 | -2.18 ± 4.85 | -2.20 ± 3.08^b^ | 0.987 |
| CARS Score | 31.11 ± 2.06 | 32.59 ± 1.85 | -1.29 ± 3.65 | -1.17 ± 3.36 | 0.919 | -2.12 ± 3.89^b^ | -3.43 ± 3.87^b^ | 0.346 |
| S-M Standardized Score | 8.00 (8.00,9.25) | 8.00 (7.00,9.00) | 0.00 (0.00,0.00) | 0.00 (0.00,1.00) | 0.460 | 0.00 (0.00,0.50) | 0.00 (0.00,1.00)^b^ | 0.184 |
| GSRS Score | 22.50 (19.75,26.25) | 21.50 (19.00,25.25) | 0.00 (-2.50,3.00) | -2.00 (-4.00,4.00) | 0.790 | -1.00 (-3.50,3.50) | -3.00 (-5.00,3.00) | 0.352 |

^b^P<0.05 compared with the Baseline.

## Table S3. Comparison of the improvement in the scores of children with ASD and a baseline CARS score of <30 between the two groups after 8 and 16 weeks of intervention [$\bar{x}$ ± *s*/M(Q1,Q3), full analysis set]

| **Scale** | **Baseline (0 d)** | | **Difference from baseline after 8 weeks of treatment** | | | **Difference from baseline after 16 weeks of treatment** | | |
| --- | --- | --- | --- | --- | --- | --- | --- | --- |
|  | **Placebo group(n=10)** | **BF839 group(n=15)** | **Placebo group(n=10)** | **BF839 group(n=14)** | **P value** | **Placebo group(n=10)** | **BF839 group (n=14)** | **P value** |
| ABC Total Score | 34.20 ± 4.26 | 41.40 ± 3.95 | -10.30 ± 5.79^b^ | -7.57 ± 18.79 | 0.617 | -13.20 ± 10.70^b^ | -11.14 ± 9.92^b^ | 0.633 |
| Sensory | 5.50 ± 1.64 | 7.67 ± 0.93 | 0.60 ± 4.99 | -0.64 ± 7.06^b^ | 0.638 | 0.60 ± 3.86 | -0.07 ± 6.08^b^ | 0.762 |
| Relating | 8.70 ± 1.56 | 7.13 ± 0.91 | -2.60 ± 3.75 | 0.07 ± 5.42 | 0.194 | -2.60 ± 4.52 | 0.64 ± 4.16 | 0.083 |
| Body and object use | 1.90 ± 1.02 | 5.60 ± 1.31 | -0.60 ± 3.31 | -1.71 ± 4.97 | 0.544 | -0.90 ± 3.35 | -3.64 ± 3.41^b^ | 0.063 |
| Language | 10.60 ± 1.93 | 11.40 ± 1.41 | -4.50 ± 3.03^b^ | -1.29 ± 5.48 | 0.108 | -5.40 ± 3.20^b^ | -3.14 ± 5.00^b^ | 0.224 |
| Social and self help | 7.50 ± 1.35 | 9.60 ± 1.27 | -2.30 ± 3.68 | -2.07 ± 5.55 | 0.910 | -2.90 ± 3.76^b^ | -2.00 ± 3.14^b^ | 0.530 |
| CARS Score | 23.10 ± 1.63 | 26.30 ± 0.80 | -0.80 ± 3.16 | -0.68 ± 3.89 | 0.936 | -2.80 ± 3.22^b^ | -2.96 ± 4.40^b^ | 0.921 |
| S-M Standardized Score | 9.50 (9.00,10.00) | 9.00 (8.00,9.00) | 0.00 (0.00,0.00) | 0.00 (0.00,1.00)^b^ | 0.163 | 0.00 (0.00,0.25) | 0.00 (0.00,1.00)^b^ | 0.168 |
| GSRS Score | 21.00 (18.75,26.25) | 20.00 (18.00,24.00) | -2.00 (-3.25,0.75) | 1.50 (-2.25,4.50) | 0.083 | -1.50 (-5.00,1.75) | -1.00 (-4.00,3.25) | 0.444 |

^b^P<0.05 compared with the Baseline.
